# Fire disturbance effects on plant taxonomic and functional β‐diversity mediated by topographic exposure

**DOI:** 10.1002/ece3.8552

**Published:** 2022-01-25

**Authors:** Mehdi Abedi, Reza Omidipour, Seyed Vria Hosseini, Khadijeh Bahalkeh, Nicolas Gross

**Affiliations:** ^1^ 41616 Department of Range Management Faculty of Natural Resources and Marine Sciences Tarbiat Modares University Noor Iran; ^2^ 185152 Department of Rangeland and Watershed Management Faculty of Natural Resources and Earth Sciences Shahrekord University Shahrekord Iran; ^3^ Université Clermont Auvergne INRAE VetAgro Sup Unité Mixte de Recherche Ecosystème Prairial Clermont‐Ferrand France

**Keywords:** climate change, functional diversity, Golestan National Park, leaf traits, natural disturbance, taxonomic diversity, turnover and nestedness, α‐ and β‐diversity

## Abstract

Although the diversity–disturbance relationship has been extensively studied, the differences in responses of taxonomic vs. functional diversity to natural disturbances (i.e., fire) call for an improved understanding of this relationship. Here, we investigated how fire disturbance influenced plant taxonomic and functional diversity in Golestan National Park, in northeastern Iran. We evaluated the response of α‐ and β‐plant diversity considering both taxonomic and functional diversity and different β‐diversity components (i.e., turnover and nestedness) as a function of fire regime, topographic exposure, and their interactive effect. We considered different indices of functional diversity including functional richness, functional evenness, functional divergence, functional dispersion, Rao's quadratic entropy, and community‐weighted mean (CWM). Functional diversity indices were computed using four leaf traits related to species growth strategy and fire response including leaf thickness and leaf length, specific leaf area (SLA) and leaf dry matter content (LDMC).

Taxonomic and functional diversity had contrasting response to fire disturbance. Fire significantly decreased taxonomic α‐diversity similarly in both north and south exposures. β‐diversity increased in south exposures but decreased in north exposures. Fire decreased functional richness, increased CWM of SLA, and decreased CWM of LDMC. In contrast, abundance‐weighted metrics of functional diversity (functional evenness, functional divergence, functional dispersion, Rao's quadratic entropy) were not impacted by fire disturbance. Finally, the main contributors to heterogeneity were driven by a fire × exposure interaction, suggesting that fire disturbance interacts with topographic exposure.

Our results suggest that taxonomic and functional α‐ and β‐diversity have contrasting responses to fire illustrating the need to consider both dimensions to understand how disturbance impacts plant communities. At large spatial scale, species turnover and nestedness appear as essential parameters to maintain species‐rich communities in response to fire disturbance.

## Graphical abstract

We investigated how fire disturbance influenced plant taxonomic and functional diversity in Mountain steppes of Golestan National Park, in northeastern Iran. In the studied area, a fire occurred in the late summer of 2013. Our results suggest that taxonomic and functional α‐ and β‐diversity have contrasting responses to fire illustrating the need to consider both dimensions to understand how disturbance impacts plant communities. Burned (left) and control (right) sites in the south exposure of study site.

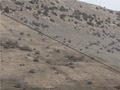

## 1 INTRODUCTION

Fire is known as one of the main natural disturbances impacting plant communities and ecosystems (Bowd et al., 2018). Many ecosystems have evolved with fire during their history resulting in fire‐adapted and fire‐dependent ecosystems (McLauchlan et al., 2020). However, the frequency and intensity of fires may significantly increase with climate change (Kasischke et al., 1995; Lima et al., 2018; Madadgar et al., 2020; Polley et al., 2013), which may have important consequences for biodiversity and the functioning of terrestrial ecosystems (Grimm et al., 2013; Hurteau et al., 2014; Omidipour et al., 2021). In the Middle East, climate change is predicted to increase temperature and decrease precipitation (Abolverdi et al., 2014; Evans, 2010; Roshan et al., 2013) which may lead to increase fire occurrence. A higher risk of fire disturbance may not only impact fire‐prone habitats but are also expected to increase in historically fire‐free habitats (Hart et al., 2019). However, little is known about fire‐free habitat response to fire disturbance. Answering this question may provide useful information in order to design appropriate management practices to protect and restore ecosystems under global change.

A large body of evidence suggests that fire has pronounced effects on plant diversity (Bowman et al., 2016; Krawchuk et al., 2009; Pausas & Ribeiro, 2013). For instance, fire can drive inter‐ and intraspecific phenotypic and genetic divergences (Gómez‐González et al., 2011; Hernández‐Serrano et al., 2013) through associating with the diversification of plant lineages (Bytebier et al., 2010; He et al., 2012, 2019). Vegetation response to fire may largely depend on plant adaptation to fire. Plant adaptation to fire includes traits related to post‐fire recruitment and growth strategies (Pausas et al., 2004). For example, fire occurrence is known to increase the prevalence of annual plants that have a lower leaf dry matter content (LDMC) and higher specific leaf area (SLA) than perennial plants (Keeley et al., 2011). Other leaf traits such as leaf thickness are related to resistance to fire disturbance (Fernandes et al., 2008; Keeley et al., 2011), while higher leaf length and LDMC likely influence flammability and fire risk (Alam et al., 2020; Lavorel et al., 2007; Simpson et al., 2016). In addition, higher specific leaf area (SLA) in shrub species is associated post‐fire resprouting (Anacker et al., 2011; Sakschewski et al., 2015). The effect of plant functional traits on plant response to fire has been widely studied in fire‐prone habitats, while there are very few studies in historically fire‐free habitats. Are plant species “preadapt” to fire in fire‐free habitat? Resprouting, flammability and germination responses to smoke have probably evolved in both fire‐prone and fire‐free areas (Bond & Midgley, 1995, 2003; Calitz et al., 2015; Pierce et al., 1995). Considering plant traits and their diversity together with a change in taxonomic diversity may increase our understanding of how plant communities will respond to fire, especially in fire‐free habitats.

Fire impact on plant communities is modulated by fire intensity (Heydari et al., 2017, 2020; Keeley, 2003, 2009). Fire intensity depends on fuel availability (quantity), but also fuel quality (e.g., fuel moisture content, Xue et al., 2018). Exposure is a key topographical factor known to influence fire intensity by modifying fuel quantity and quality (Estes et al., 2017). For instance, topographic exposure influences solar radiation and soil moisture, and vegetation biomass (Billings, 1974) leading to marked differences in fuel quantity and quality between exposures (Holden et al., 2009). A trade‐off between fuel quantity and quality exists between different topographic exposures. In Northern Hemisphere, north exposures have on average more vegetation biomass than south exposures especially in water‐limited ecosystems (Bahalkeh et al., 2017; Estes et al., 2017). However, vegetation on north exposures dries slowly and retains more moisture, making them less flammable than south exposures. Therefore, different topographic exposures may largely mediate the effect of fire disturbance on plant diversity.

Fire may alter plant diversity at local and large spatial scales (e.g., landscape). β‐diversity is defined as the variation in species composition across communities (Anderson et al., 2011; Tuomisto, 2010) and can be quantified using both taxonomic (taxonomic β‐diversity; Whittaker, 1960) and functional trait diversity (functional β‐diversity; Ricotta & Burrascano, 2008; Swenson et al., 2011; Villéger et al., 2013). β‐diversity may vary across sampling units due to species gain/loss (i.e., change in species richness across sampling units, hereafter species nestedness) or may vary due to species replacement (i.e., change in species composition that can be independent from change in richness, hereafter species turnover) (Baselga, 2010). The contribution of each of these β‐diversity components helps to understand the drivers of vegetation change through space (Heydari et al., 2019). For example, a change in nestedness could highlight a strong effect of fire on plant diversity leading to potentially important species loss. In contrast, a change in turnover could inform us on the ability of vegetation to cope with fire by recruiting new species adapted to fire. Both taxonomic and functional β‐diversity have been used to evaluate different vegetation responses to disturbance on plant communities (Feizabadi et al., 2021; Moradizadeh et al., 2020). For instance, Heydari et al. (2017) have shown how fire intensity impacts soil seed bank and aboveground vegetation and highlighted the role of species turnover as the main contributor to β‐diversity in response to fire disturbance. Partitioning diversity into their α‐ and β‐diversity for both taxonomic and functional diversity components could improve our ability to predict how fire impacts plant diversity within and across communities (Bishop et al., 2015).

In this study, we aimed to assess the effect of fire disturbance and different topographic exposures on taxonomic and functional diversity in the mountain steppes of Iran. Mountain steppes in Golestan National Park are species‐rich habitat and include a wide variety of perennial species such as *Onobrychis cornuta*, *Festuca valesiaca*, *Stipa lessingiana*, *Poa densa*, *P*. *bulbosa*, *Bromus tomentellus*, and *Koeleria macrantha* exhibiting resprouting strategies to disturbance (Bahalkeh et al., 2021). These habitats have low wildlife grazing (mostly Urial sheep, *Ovis ammori*), and fire is not a common disturbance (Abedi et al., 2018; Bureau of Environment in Golestan Province, 2014). However, fire event has increased recently in the region (Bahalkeh et al., 2021; Jahdi et al., 2016) and may become an important driver of vegetation dynamics in the future. Understanding how plant species and functional diversity respond to fire disturbance across contrasted spatial scale and under different topographic exposures may help in establishing effective management plans for the biodiversity conservation in this IUCN natural reserve. We addressed to answer the following questions:

1. What are fire effects on the taxonomic and functional α‐ and β‐diversity and how these effects are related to the different topographic exposure?
2. Does the contribution of taxonomic and functional β‐diversity components (turnover vs. nestedness) vary with fire disturbance and topographic exposure?

## 2 MATERIALS AND METHODS

### 2.1 Description of the study area

Mountain steppes occur throughout the North East of Iran including Golestan National Park where calcareous‐rich soils developed mostly during and after Upper Precambrian and Jurassic (Akhani, 1998; Memariani et al., 2016). Today, this steppe includes calcareous species‐rich grasslands dominated by thorn‐cushions, perennial grasses, and forbs (Bahalkeh et al., 2018). Golestan National Park has been under conservation policy since 1956 (Golestan National Park report). Different parameters such as wildlife grazing, drought, disturbance by wild boar, and fire are the main drivers of vegetation dynamic in this habitat (Akhani, 1998; Bahalkeh et al., 2021; Safaian et al., 2005).

The study was conducted in Alme‐Gharatikan located in Golestan National Park, one of the most important National Park and Biosphere reserves in Iran and located in latitude 37°19′52.42″N and longitude 56°6′0.96″E. Altitude ranges between 1700 and 2000 m a.s.l. Mean long‐term annual precipitation and temperature are 550.2 mm and 9.8°C, respectively. Alme‐Gharatikan area is located in the central part of Golestan National Park where a well‐developed community of rich herbaceous species is dominated by small cushion plants (Bahalkeh et al., 2018, 2021). This habitat is known as a transition region wherein changes to Artemisia steppes in the lowlands and toward higher mountain mixed with *Acer monspessulanum*, *Juniperus excelsa*, and other shrub species. The total vegetation often covers up to 40% of the ground (53% in our study sites) (Bahalkeh et al., 2018).

Fire events have increased in the recent years in most of Golestan National Park due to both drought and human, that is, fire frequency increased by a factor of 5 in the last ten years (Bureau of Environment in Golestan Province, 2014). In the studied area, a fire occurred in the late summer of 2013 and burned around 400 ha. Before this date, no fire has been reported in studied area since early records starting back to 1953 (Bureau of Environment in Golestan Province, 2014). The prolonged absence of fire from our study area was further confirmed by the presence of slow‐growing shrubs—*Juniperus excelsa* and *Acer monspessulanum*—which are killed by fire (Bahalkeh et al., 2021; Figure S1).

### 2.2 Vegetation sampling

Vegetation sampling was conducted in July 2015, two years after fire occurrence. In an area of 100 ha, we selected two large burned (fire treatment) and unburned sites (control treatment) of at least 50 ha. Both sites were located at the same topographic condition's exposures. In each fire and control site, two southern and northern exposures were selected (more details in Bahalkeh et al., 2021). Two selected exposures represent different drought stress conditions, when northern exposure (hereafter north exposure) shows low stress and southern exposure (hereafter south exposure) shows high stress according to previous studies in these mountain grasslands (Al Hayek et al., 2015). South exposure has shallow stony soils (stone cover = 59.3 ± 1.7, *n* = 20 plots 4 × 4 m) in drier and convex topography, and north exposure has deeper and developed soils (stone cover = 0.6 ± 0.1, *n* = 20 plots 4 × 4 m) in wetter and concave topography. There are no differences between soil texture and stone covers of fire and control sites in each exposure (Bahalkeh et al., 2021). In total, four sites were selected for the study including fire and control in two exposure treatments (SF and SNF = southern exposure with and without fire, respectively; NF and NNF=northern exposure with and without fire, respectively). Each of the four sites was sampled using 20 plots located within an area of approx. 25 ha. In each site, we randomly established 20 plots (4 × 4 m) (Arzani & Abedi, 2015) for a total of 80 plots (2 fire treatments × 2 topographic exposures × 20 plots = 80 plots). In July 2015, the cover of all vascular species present in the plots was recorded, and then, species cover for all plots was calculated. Plants were identified at the species level.

### 2.3 Functional traits

Four key leaf functional traits known to be associated with fire disturbance were selected for analysis. For the most abundant species (56 species of total 60 species in the study area), we measured four leaf traits: specific leaf area (SLA), leaf dry matter content (LDMC), leaf thickness (LTH), and leaf length (LL). These traits were selected because they well reflect plant growth strategies, and are good candidate traits to reflect species response to fire but also how species influence vegetation effect on fire risk. SLA and LDMC well reflect plant growth rate with species high SLA and low LDMC having higher growth rates than species with low SLA and LDMC. Our hypothesis is that fire increases SLA and decreases LDMC because fire could increase the abundance of fast‐growing and ruderal species compared with perennial species (Davies et al., 2009; Khaled et al., 2006; Rhodes et al., 2010). LTH is known to reflect species resistance against fire (Fernandes et al., 2008; Keeley et al., 2011), and therefore, we expect that fire increased LTH within plant communities. Finally, high LL and LDMC are associated with the dominance of grass species in dry areas (Gross et al., 2013) and positively influence fire occurrence and intensity (Alam et al., 2020; Lavorel et al., 2007; Simpson et al., 2016). We acknowledge that we may have discarded other important traits related to plant response to fire such as traits related to plant regeneration (seed mass, seed number, resprouting strategies) or chemical traits (leaf nitrogen content). However, our trait selection included important traits related to plant forms and functions (Díaz et al., 2016) and to plant strategy (*sensu* CSR strategies of Grime, 1973) and is known correlated with important plant morphological and chemical attributes. For instance, higher specific leaf area (SLA) positively correlated with nitrogen in leaves (Díaz et al., 2016; Wright et al., 2004), promoted post‐fire resprouting in shrubs (Anacker et al., 2011; Sakschewski et al., 2015), and differentiate annual from perennials plant species in herbaceous communities (Keeley et al., 2011). We collected 2 mature leaves from 5 individuals per species (560 leaves in total) randomly selected in the control areas (two no‐fire areas). Leaf traits were measured in the laboratory using leaf dry and fresh weight, LTH with a micrometer, and leaf area according to standard protocols (Cornelissen et al., 2003).

### 2.4 Taxonomic diversity calculation

To quantify species diversity, we used α‐ and β‐diversity indices, defined as the number of species within and among sampling plots, respectively. The α‐diversity and β‐diversity were calculated based additive partitioning method (Crist et al., 2003; Lande, 1996) in which total species diversity (γ‐diversity) additively divided into within (α‐diversity) and among (β‐diversity) samples (γ‐diversity = α‐diversity + β‐diversity). The additive partitioning method focusses on the same currency (species richness) across scales and provides a straightforward methodology to study diversity pattern through space (Lande, 1996). In detail, the number of species in each plot and each of the four sites was calculated as α‐ and γ‐diversity, respectively. Then, the β‐diversity was obtained by subtracting α‐diversity from γ‐diversity (β‐diversity = γ‐diversity − α‐diversity). It must be noted that α‐diversity and β‐diversity were calculated at the plot level. Therefore, we obtained 20 data points in each treatment for a total of 80 data points.

### 2.5 Functional diversity calculations

We calculated the community‐weighted mean (CWM) for each trait (Violle et al., 2007), obtained by summing the product of the relative cover and trait values of each species. For functional diversity, we considered Rao's quadratic entropy index (Rao, 1982) which is one of the most common functional diversity indices (Tahmasebi et al., 2017). Rao index was calculated per plot as the abundance‐weighted dispersion of trait values within a given community. We also computed other complementary multi‐trait functional indices such as functional richness (FRic), functional evenness (FEve), and functional divergence (FDiv) using methods developed by Mason et al. (2005) and extended by Villéger et al. (2008). FRic is the volume of multidimensional trait space occupied by all species in a community. FEve reflects the regularity of the distribution of trait values and abundance within a community. FDiv summarizes the proportion of total abundance supported by species with the most extreme trait values within a community (See Mouillot et al., 2013 for detailed presentation of each index). The different components of functional diversity indices were calculated using the “FD” package (Laliberté et al., 2015). We did not consider the functional dispersion (FDis, Laliberté & Legendre, 2010) in our study as FDis index was highly correlated with RaoQ (*r* = .954 and *p* < .001).

### 2.6 Partitioning taxonomic and functional diversity

We partitioned taxonomic and functional β‐diversity (Baselga, 2010; Villéger et al., 2013) into a turnover and nestedness components. To do so, we used pairwise dissimilarity derived from the Sorensen coefficient in which total taxonomic and functional dissimilarities are decomposed into components of taxonomic and functional turnover and nestedness. The partitioning analysis was performed in four cases (hereafter scenarios) including comparisons between two inter‐treatments in the same exposure (NF‐NNF and SF‐SNF) and comparisons between two exposure transgressing in the same treatments (NF‐SF and NNF‐SNF). These analyses were performed at the site scale, that is, by aggregating data belonging to one site because turnover and nestedness components taxonomic and functional β‐diversity were calculated across plots (Baselga et al., 2018). Functional β‐diversity was calculated based on the four functional traits including SLA, LDMC, LTH, and LL. Both taxonomic β‐diversity and functional β‐diversity were quantified using multiple‐site taxonomic/functional Jaccard dissimilarity methods developed by Villéger et al. (2013). The taxonomic and functional β‐diversity and their components were calculated using the “betapart” package (Baselga et al., 2018).

### 2.7 Statistical analysis

To determine the species pool overlap among the studied sites (NF, NNF, SF, SNF), the Venn diagram was drawn using “ggvenn” package. In addition, we performed a taxonomic rarefaction to remove the effects of sampling effort on our results. The taxonomic rarefaction was done based on rarefy curve of vegetation matrix using species richness index (based q=0 of Hill diversity index) and relative vegetation cover in “vegan” and “iNEXT” packages.

To test the effect of fire and exposure on plant diversity, we used two‐way analysis of variance (ANOVA) with fire (fire vs. control), exposure (north vs. south), and their interaction as the fixed factors and α‐ and β‐diversity as well as different functional diversity indices as the dependent factors followed by Duncan post hoc test. This analysis was repeated for each dependent factor, separately. Before statistical analysis, normality and homogeneity of the data were tested using the Kolmogorov–Smirnov test and Levene's test, respectively. All analyses were performed in the R version 3.5.2 (R Core Team, 2018).

## 3 RESULTS

In total, 60 species from 20 plant families were recorded. The most abundant families were Asteraceae (16.1%), Poaceae (14.2%) followed by Lamiaceae (12.5%), and the most abundant species from northern exposure with fire (NF) were *Stipa lessingiana* (6.71%), *Onobrychis cornuta* (4.33%), *Festuca valesiaca* (2.81%), *Serratula latifolia* (2.76%), *Verbascum speciosum* (1.98%), *and Crucianella sintenisii* (1.92%). In the southern exposure with fire (SF), *Serratula latifolia* (10.76% cover), *Phlomis cancellata* (5.55%), *Festuca valesiaca* (3.76%), *Stipa lessingiana* (3.5%), *Onobrychis cornuta* (3.25%), *and Centaurea virgate* (2.96%) were the most frequent species. In the northern exposure without fire (NNF), *Onobrychis cornuta* (17.28%), *Festuca valesiaca* (11.48%), *Stipa lessingiana* (8.99%), *Serratula latifolia* (6.19%), *Koeleria macrantha* (6.12%), *and Cephalaria microcephala* (5.91%) were the most frequent species. In the southern exposure without fire (SNF), *Stipa lessingiana* (6.71%), *Onobrychis cornuta* (4.32%), *Festuca valesiaca* (2.81%), *Serratula latifolia* (2.76%), *Verbascum speciosum* (1.98%), *and Crucianella sintenisii* (1.92%) were the most frequent species.

Perennial grasses followed by perennial herbs and shrubs were the main dominant plant functional groups in the NNF site. Although perennial herb and perennial grass cover were strongly reduced by fire, they were still the most dominated functional groups in NF site (Table S1). In the SNF site, both perennial herbs and perennial grasses were dominated while fire decreased the cover of perennial grasses at the SF site (Table S1).

**FIGURE 1:**
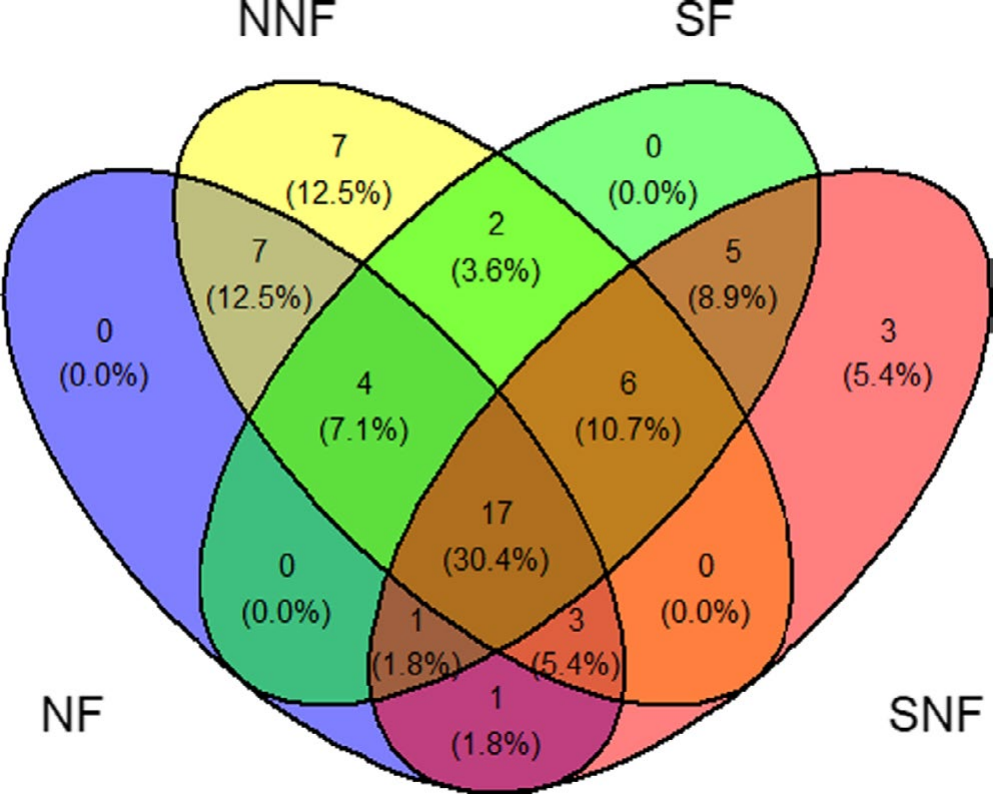
The Venn diagram of overlap in the species pools under fire treatments and exposures. NF and SF: northern and southern exposures with fire, respectively, NNF and SNF: northern and southern exposures without fire, respectively. The numbers outside and inside of the parenthesis are the number and percent of total species in each case, respectively

The Venn diagram showed that the highest unique species were observed in the northern and southern exposures without fire sites (7 and 3 species, respectively), while there was no unique species with fire (Figure 1). In addition, there were 31 and 29 common species between sites in the same topographic exposures, that is, NF‐NNF and SF‐SNF, respectively, and 17 species were shared across all sites (Figure 1).

**FIGURE 2:**
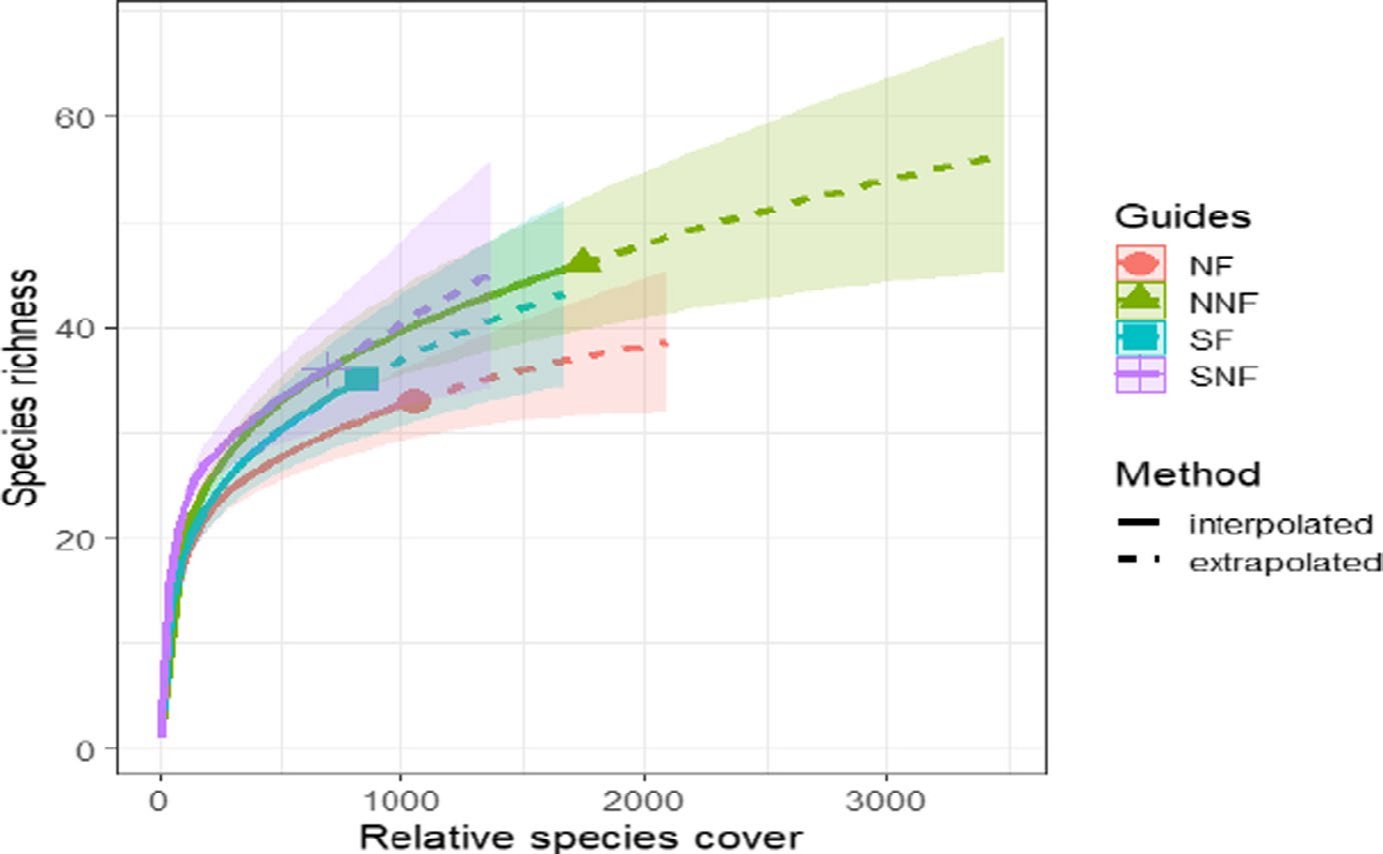
Taxonomic rarefaction curve‐based relative species cover under fire treatments and exposures. NF and SF: northern and southern exposures with fire, respectively, NNF and SNF: northern and southern exposures without fire, respectively

The taxonomic rarefaction results showed little evidence for asymptotic curve (Figure 2), indicating that increasing further the sampling effort would continue to slightly increase the number of species sampled. In addition, the species richness in sites without fire (NNF and SNF) was greater than those found on fire sites (NF and SF). Note that NNF site had much higher relative cover than the other sites.

### 3.1 Fire and exposure effects on taxonomic α‐ and β‐diversity

**FIGURE 3:**
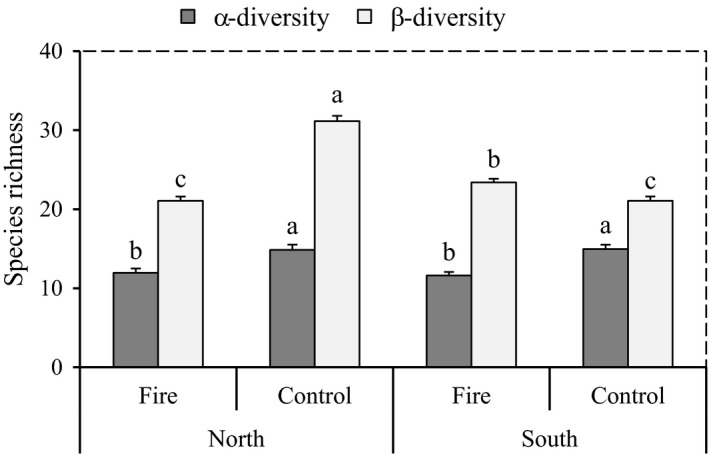
The results of comparing mean (mean ± SE) for taxonomic α‐ and β‐diversity separately, under fire treatment and exposure. Different letters indicate a significant difference between each diversity indices under fire treatment and exposure at *p* < .05

Taxonomic α‐diversity decreased with fire (*F*‐value = 30.34, *p*‐value > .0001), and this effect was not influenced by exposure (exposure *p*‐value = .826; fire–exposure interaction *p*‐value = .963; Table 1). Species richness peaks in control areas at 14.85 species and 14.95 species in northern and southern exposures without fire (NNF and SNF), respectively (Figure 3).

**TABLE 1:** The results of two‐way ANOVA for the effects of fire, exposure, and their interaction on taxonomic α‐ and β‐diversity. *R* ^2^ indicates the percent variation explained in models. The significant effects are indicated in bold

Source of variation	α‐diversity			β‐diversity	
	*df*	*F*‐value	*p*‐value	*F*‐value	*p*‐value
Fire	1	30.34	**>.0001**	46.65	**>.0001**
Exposure	1	0.049	.826	46.65	**>.0001**
Fire * Exposure	1	0.157	.963	120.39	**>.0001**
*R* ^2^	0.278			0.738	

In contrast to α‐diversity, β‐diversity was significantly affected by all our treatment including fire (*F*‐value = 46.65, *p*‐value > .0001), exposure (*F*‐value = 46.65, *p*‐value > .0001), and a fire–exposure interaction (*F*‐value = 120.39, *p*‐value > .0001; Table 1, Figure 3). Fire decreased and increased β‐diversity in northern and southern exposures, respectively. The highest amount of β‐diversity was found in the NNF (31.15 ± 0.67 species). The lowest β‐diversity was found either in control areas on south exposure and areas with fires on north exposure (21.05 ± 2.48 and 21.05 ± 2.5, respectively).

### 3.2 Fire and exposure effects on single‐ and multi‐trait functional diversity indices

**FIGURE 4:**
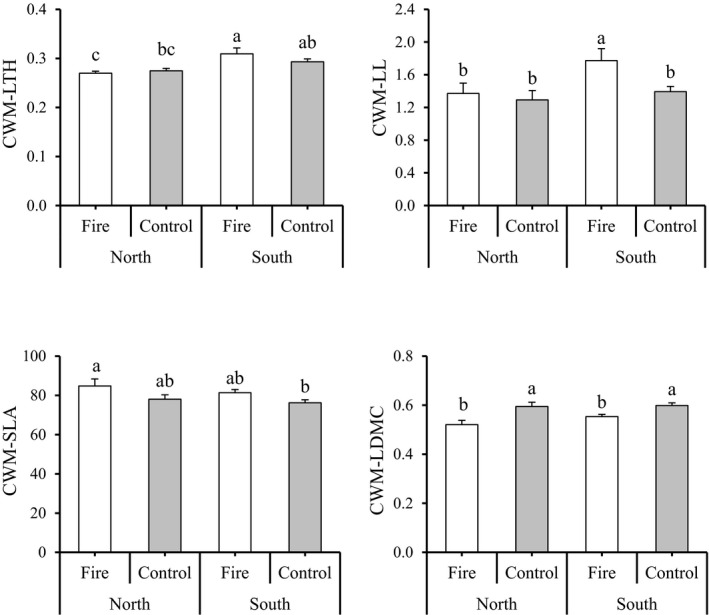
The results of comparing mean (± SE) for single trait‐based index of functional diversity under fire treatment (fire vs. control) and exposure (north vs. south). Different letters indicate a significant difference at *p* < .05

Fire significantly impacts community‐weighted mean (CWM) traits values. Fire increased CWM‐SLA (*F*‐value = 6.26, *p*‐value < .0001) and decreased CWM‐LDMC (*F*‐value = 16.81, *p*‐value < .0001). In contrast, CWM‐LTH was not significantly influenced by fire (*F*‐value = 0.606, *p*‐value = .439; Table 2). In addition, CWM‐LTH (*F*‐value = 15.79, *p*‐value < .0001) and CWM‐LL (*F*‐value = 4.66, *p*‐value = .034) were significantly affected by exposure (Table 2). CWM‐LTH was greater in south versus north exposure. Fire increased CWM‐LL on southern exposure (*F*‐value = 3.85, *p*‐value = .05) (Figure 4). Finally, we did not detect any significant interaction between fire and exposure on CWM values.

**TABLE 2:** Fire, exposure, and their interaction effects on the different components of functional diversity (single‐ and multi‐trait indices). *R^2^* indicates the percent variation explained in models. The significant effects are indicated in bold

Treatment	CWM‐LTH			CWM‐LL		CWM‐SLA		CWM‐LDMC	
	*df*	*F*	*p*‐value	*F*	*p*‐value	*F*	*p*‐value	*F*	*p*‐value
Fire	1	0.606	.439	3.85	.**05**	6.26	.**014**	16.81	**<.0001**
Exposure	1	15.79	**<.0001**	4.66	.**034**	1.21	.275	1.61	.208
Fire * Exposure	76	2.12	.150	1.66	.201	0.11	.739	0.98	.325
*R* ^2^	0.196			0.118		0.091		0.203	

Treatment	FRic			FEve		FDiv		RaoQ	
	*df*	*F*	*p*‐value	*F*	*p*‐value	*df*	*F*	*p*‐value	*F*
Fire	1	6.34	.**014**	0.007	.933	1	6.34	.**014**	0.007
Exposure	1	0.89	.347	4.54	.**036**	1	0.89	.347	4.54
Fire * Exposure	76	1.77	.187	1.12	.294	76	1.77	.187	1.12
*R* ^2^	0.106			0.069		0.019		0.241	

**FIGURE 5:**
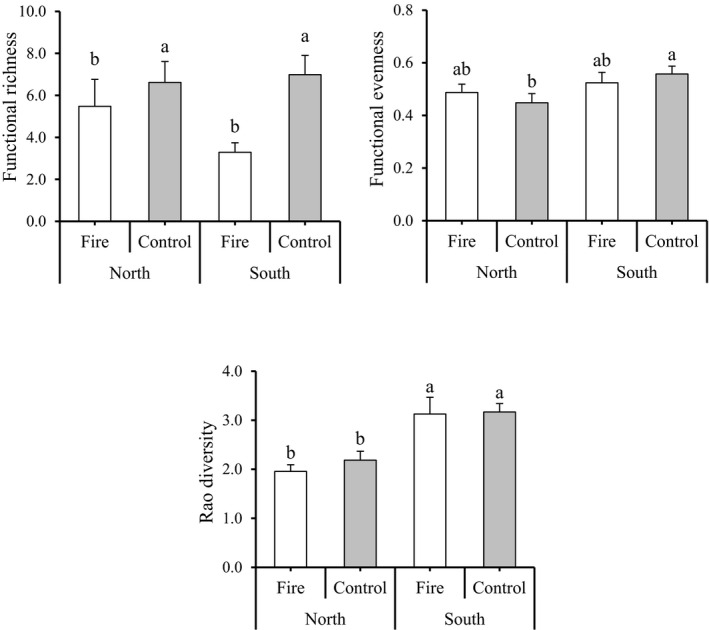
The results of comparing mean (mean ± SE) for multiple trait‐based indices of functional diversity under fire treatment (fire vs. control) and exposure (north vs. south). Different letters indicate a significant difference at *p* < .05

Fire significantly decreased FRic independently from exposures (*F*‐value = 6.34, *p*‐value = .014; Table 2) (Figure 5) while other abundance‐weighted functional diversity components (FEve, FDiv, and RaoQ) were not significantly influenced by fire (all *p*‐value > .05) (Table 2). Exposure significantly effects on the FEve (*F*‐value = 4.54, *p*‐value = .036) and RaoQ (*F*‐value = 23.57, *p*‐value < .0001), while FRic and FDiv were not significantly affected by exposure (Table 2). FEve and RaoQ were higher in south compared with the north exposure (Figure 5).

### 3.3 Partitioning taxonomic and functional β‐diversity into turnover and nestedness

**FIGURE 6:**
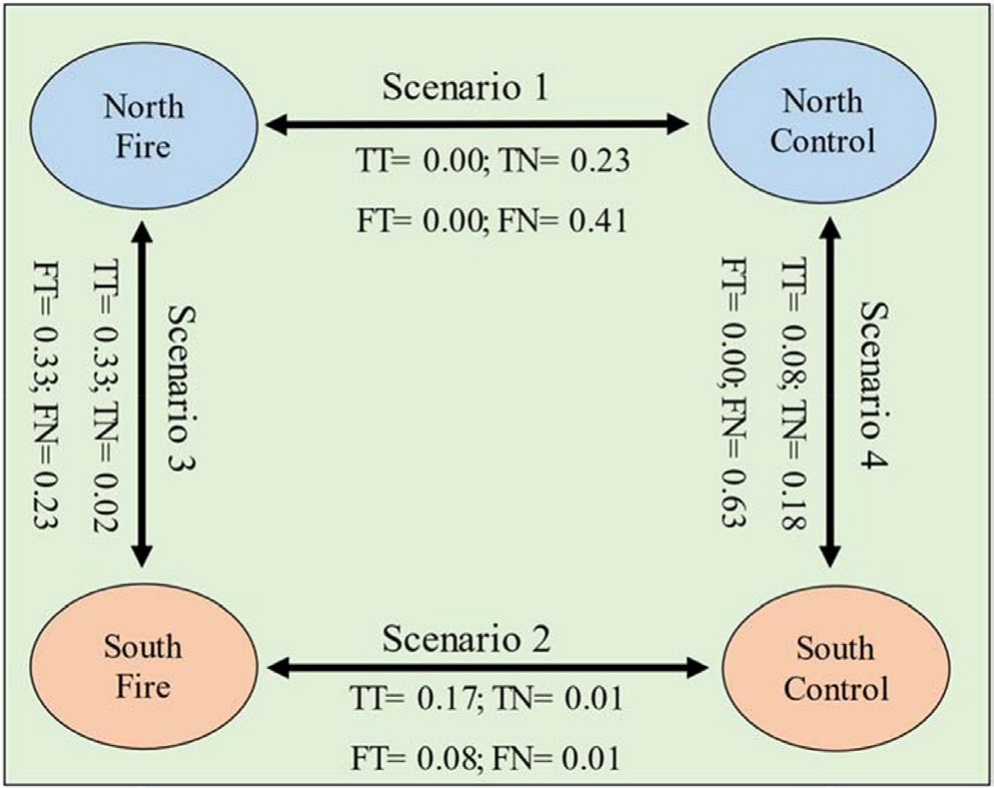
Partitioning taxonomic and functional β‐diversity into turnover and nestedness components in a pairwise comparison in four scenarios including between the same exposure (NF and NNF) and same treatment (NF‐SF and NNF‐SNF). TT and FT are taxonomic and functional turnover, and TN and FN indicate taxonomic and functional nestedness

Partitioning β‐diversity into turnover and nestedness components showed similar results in the same exposure for both taxonomic and functional. On north exposures, heterogeneity due to fire was explained by species gain and loss (nestedness) (Figure 6). In contrast, differences in south exposures due to fire were mainly explained by species turnover for both taxonomic (94%) and functional (89%) β‐diversity (Figure 6). Also, between the same treatments, species turnover was the main contributor to taxonomic β‐diversity (94%) in fire areas between NF‐SF, while in the functional β‐diversity, turnover (58%) had slightly more than half contribution compared with nestedness (42%). A similar pattern was observed in control areas between NNF and SNF sites in which nestedness plays a greater role in taxonomic (69%) and functional (100%) β‐diversity (Figure 6).

## 4 DISCUSSION

### 4.1 Fire effects on taxonomic diversity

In our study, fire effect on taxonomic α‐diversity was negative and independent from exposure. The negative effect of fire contrasted with results generally observed with prescribed burning (Pastro et al., 2014) and non‐prescribed fire on plant diversity (Heydari et al., 2017). Fire has been shown to increase plant diversity because: (i) fire decreases competition among plants and stimulates soil seed banks, especially in fire‐prone ecosystems (Gómez‐González et al., 2011; Hernández‐Serrano et al., 2013); and (ii) plants in recently burnt vegetation are generally smaller, and thus, it increases the density of individual plant per unit area, therefore increasing the chance to detect more species in a given sampling unit (Gotelli & Colwell, 2001). However, our taxonomic rarefaction results clearly confirmed the negative effect of fire on taxonomic α‐diversity where plant richness was greater in non‐fire sites (i.e., NNF and SNF). This finding may be explained by the fire history of the vegetation in Golestan National Park where vegetation may not be adapted to fire. Several perennial forbs such as *Koeleria macrantha*, *Cephalaria microcephala*, *Centaurea kotschyi*, *Ephedra major*, *Opopanax hispidus*, *Euphorbia buhsei*, *Salvia atropatana*, *Cirsium bornmuelleri*, *Galium verum*, *Pimpinella tragium*, *Alyssum tortuosum*, *Helichrysum oocephalum*, *Inula oculus*‐*christi*, *and Mesostomma kotschyana* were not observed anymore in fire treatments, suggesting that fire may have removed those species from the local community. Therefore, fire occurrence in this historically fire‐free habitat likely resulted in decline species richness by removal of perennial species not adapted to fire disturbance. We recognize that our short‐term sampling two years after burning event does not allow quantifying whether or not those sensitive species to fire could recolonize burnt areas. Long‐term monitoring might be needed to evaluate the recolonization time of these species and how fire frequency and intensity could constitute a threat to fire‐sensitive species under climate change in Golestan Park.

Fire differently impacts plant diversity at larger spatial scale as we observed a significant effect of fire on β‐diversity that varied with exposure. Fire disturbance significantly increased and decreased β‐diversity in SF and NF, respectively. Bahalkeh et al. (2021) showed that north exposure is characterized by higher fire severity than south exposure due to higher available biomass and larger cushion size. Fire with higher severity is often expected to homogenize species composition (Pausas & Verdú, 2008) resulting in a decrease in β‐diversity on northern slopes. Similarly, harsher environment conditions in the south exposure and lower fire severity may result in a higher β‐diversity on southern slopes. Given that fire similarly decreased α‐diversity in both north and south exposures, therefore, β‐diversity is likely to be a better proxy to indicate disturbance effects of plant communities’ diversity and composition in different topographical exposure. As our studied sites were fire‐free ecosystems and did not face a long fire history, the southern exposure may be less influenced against fire and diversity loss due to harsher conditions (i.e., low soil moisture) compared with the north exposure site. However, the unfavorable condition in south exposure also could affect post‐fire regeneration (Arnan et al., 2007), resulting in lower regeneration in the south than in the north exposures. Conversely on north exposure, a higher ecosystem productivity coupled with the milder environmental conditions may decreased recovery time after fire disturbance compared with south exposures (Calvo et al., 2012).

### 4.2 Fire effects on functional diversity

CWM‐leaf traits were more affected by the short‐term effect of fire than exposure. Two years after the fire event, we observed lower CWM‐LDMC and higher CWM‐SLA in fire areas compared with control. This result is in line with the hypothesis that fire disturbance may increase the abundance of fast‐growing and ruderal species such as annuals species, especially in the early years after fire occurrence (Davies et al., 2009; Khaled et al., 2006; Rhodes et al., 2010). For example, NF led to appearance of new annual species (such as *Aegilops tauschii* Coss. and *Scabiosa rotata* M. Bieb.) and biennial species (such as *Onosma dichroantha* Boiss.) while several perennial forbs (such as *Helichrysum oocephalum* Boiss. and *Ephedra major* Host.) and perennial grasses (such as *Elymus hispidus* (Opiz) Melderis. and *Koeleria macrantha* (Ledeb.) Schult.) were not observed anymore in burnt areas. Higher SLA values positively correlated with higher photosynthesis and growth rates (Westoby et al., 2002; Wright et al., 2004). Therefore, it can be concluded that fire disturbance promotes the abundance of small‐statured fast‐growing species, that is, R‐strategy with higher palatability and productivity (Grime, 1973).

Fire also reduced FRic while poorly explained other functional trait diversity indices weighted by species abundance (i.e., FEve, FDiv, and Rao indices). This result indicated that, contrary to functional richness, abundance‐weighted indices may be driven by other ecological variables than fire (i.e., environmental productivity). Also, the negative effect of fire disturbance on trait richness (FRic) indicates that fire had filtered out some species with extreme trait value (i.e., functionally rare species), therefore reducing trait space observed at the community level, for example, NF compared with NNF, eliminate *Silene latifolia* Poir. and *Opopanax hispidus* (Friv.) Griseb. which had the lowest and highest plant leaf thickness (0.08 and 0.93 mm, respectively) and in SF compared with SNF, eliminated species with higher LDMC such as *Scabiosa rotata* M. Bieb., *Berberis vulgaris* L., and *Acantholimon scirpinum* Bunge‐Antheis (LDMC (g/g) = 0.64, 0.64, and 0.82, respectively). Similar results were reported for other disturbance types (i.e., grazing) in alpine meadows on the Qinghai–Tibetan Plateau (Li et al., 2019). Generally, fire is known as an evolutionary pressure that could alter plant trait richness (Keeley et al., 2011). Fire decreased trait richness likely because some trait combinations may be not adapted to fire (Buhk et al., 2007) and are disadvantaged when the fire occurred. In Golestan Park, the concomitant negative effects of fire disturbance on functional and taxonomic richness suggest that fire homogenized plant communities by filtering out functionally rare and unique species (Violle et al., 2017).

### 4.3 Partitioning taxonomic and functional β‐diversity

To consider the contribution of β‐diversity components, we used the four scenarios including comparisons of between two inter‐treatments in the same exposure (Scenario 1: comparison between NF and NNF; Scenario 2: SF‐SNF) and comparisons between two exposures in the same treatment (Scenario 3: NF‐SF; Scenario 4: NNF‐SNF) (Figure 6). In Scenario 1 (i.e., taxonomic and functional β‐diversity between NF‐NNF), both taxonomic β‐diversity and functional β‐diversity were driven by nestedness. In northern exposure, there is a wider trait space compared with southern exposure due to higher water availability (Holden et al., 2009). Higher total species richness (i.e., γ‐diversity, sum α‐ and β‐diversity) in NNF compared with SNF (46 vs. 36 species in NNF and SNF, respectively) confirms this pattern. Therefore, as almost all species occurred in the NNF site (46 of 56), β‐diversity differences between NNF site and other sites (i.e., NF and SNF sites) were much likely to be nested, because NNF included all present species in the study area. In addition, fire occurrence in north exposure resulted in the loss of several common species between control and fire sites (i.e., perennial species). Therefore, fire in north slopes led to a sub‐setting of the community composition and trait space (i.e., taxonomic and functional nestedness, respectively), enforcing nestedness between fire and no‐fire sites, with the fire‐site community being nested into the no‐fire community.

In Scenario 2 (Figure 6), turnover was the main driver of taxonomic and functional β‐diversity between SF‐SNF (the second scenario). Because of the drought effects on the southern exposure, the trait volume (trait spectrum) may be already narrowed due to a higher degree of specialization in the community (Lambers et al., 2008). Therefore, setting fire on this condition did not narrow the trait space, but led to a functional shift, and also the turnover of species with contrasted trait values rather than nestedness.

In Scenario 3 (Figure 6), β‐diversity was driven by turnover (more so for taxonomic than for functional, 94, and 58%, respectively). The fire had a narrowing effect on the trait space of the northern exposure (Pausas et al., 2004), but had a weak effect on the already narrowed (due to drought selection pressure) trait space of the southern exposure. Due to fire, the size of the trait space on the northern exposure became more similar to the size of the trait space of the community on the southern exposure, suggesting that fire and drought may have similar filtering effect on plant communities (Pausas & Verdú, 2008). However, the species that were carved away on the northern exposures were not necessarily the ones that are absent from the southern exposure, which led to turnover being the dominant driver of β‐diversity between the two exposures under fire.

Finally, in Scenario 4 (Figure 6), both taxonomic β‐diversity and functional β‐diversity were driven mainly by nestedness. Generally, the trait space on the northern exposure is wider than the southern exposure due to the selection pressure exerted by drought. North exposure also included species/functional types that could not prevail on the southern exposure because they are not adapted to the harsh dry conditions in the southern exposure (Basu et al., 2016; Holden et al., 2009). However, the drought specialists from in the southern exposure could also grow on the northern exposure because the conditions are not different enough to exclude them from north exposure. This led to a situation where the plant community from the southern exposure was nested into the community of the northern exposure.

## 5 CONCLUSION

In the present study, the simultaneous effects of fire and topographic exposure on species and functional diversity and heterogeneity were investigated. The results indicated that although fire decreased α‐diversity irrespective of topographic exposure, its effect on heterogeneity varied with exposure. Also, multi‐traits functional diversity indices (such as FEve and RaoQ) were more affected by topographic exposure, while the community‐weighted mean (CWM) of plant functional traits (such as CWM‐LL, CWM‐SLA, and CWM‐LDMC) was affected by the fire. Our results reveal that a decrease in FRic in response to fire lead to a functional homogenization of the plant communities of Golestan Park. The nestedness was the main contributor to heterogeneity between NF‐NNF and NNF‐SNF sites while turnover was the main driver of taxonomic and functional β‐diversity between SF‐SNF and NF‐SF sites, suggesting that fire disturbance interacts with drought stress. Our results provide support for the negative effects of fire disturbance on species and functional diversity in historically fire‐free habitats in a short time, and it may accelerate by climate change. Further studies are needed to understand long‐term recovery after fire disturbance of these historically fire‐free habitat. Specifically, how landscape heterogeneity could promote recolonization rates and the persistence of fire‐sensitive species across the whole of Golestan National Park need to be assessed. Should there be no threat to species, then fire and the management thereof, should be considered in the context of the functioning of the whole ecosystem and the management goals for the whole Golestan National Park. Fire clearly promotes more productive, and potentially more palatable, species on southern slopes, and could potentially be used to promote forage abundance and quality for the Urial sheep (listed as vulnerable by the IUCN) and habitat for other fauna such as birds, insects, reptiles, and small mammals.

## CONFLICT OF INTEREST

There is no conflict of interest.

## AUTHOR CONTRIBUTIONS

**Mehdi Abedi:** Conceptualization (lead); Investigation (equal); Software (lead); Supervision (lead); Validation (equal); Writing – original draft (equal); Writing – review & editing (equal). **Reza Omidipour:** Formal analysis (equal); Software (equal); Writing – review & editing (equal). **Seyed Vria Hosseini:** Investigation (equal); Resources (supporting). **Khadijeh Bahalkeh:** Data curation (equal); Investigation (equal); Writing – review & editing (equal). **Nicolas Gross:** Conceptualization (equal); Formal analysis (equal); Methodology (equal); Software (equal); Supervision (equal); Validation (equal); Writing – review & editing (equal).

## Supporting information

Supplementary Material

## Data Availability

https://doi.org/10.6084/m9.figshare.16577300.v1

## References

[ece38552-bib-0001] Abedi, M. , Zaki, E. , Erfanzadeh, R. , & Naqinezhad, A. (2018). Germination patterns of the scrublands in response to smoke: The role of functional groups and the effect of smoke treatment method. South African Journal of Botany, 115, 231–236. 10.1016/j.sajb.2017.03.010

[ece38552-bib-0002] Abolverdi, J. , Ferdosifar, G. , Khalili, D. , Kamgar‐Haghighi, A. A. , & Haghighi, M. A. (2014). Recent trends in regional air temperature and precipitation and links to global climate change in the Maharlo watershed, Southwestern Iran. Meteorology and Atmospheric Physics, 126(3), 177–192. 10.1007/s00703-014-0341-5

[ece38552-bib-0003] Akhani, H. (1998). Plant biodiversity of Golestan National Park, Iran. Stapfia, 53, 1–411.

[ece38552-bib-0004] Al Hayek, P. , Maalouf, J. , Baumel, A. , Bou Dagher‐Kharrat, M. , Médail, F. , Touzard, B. , & Michalet, R. (2015). Differential effects of contrasting phenotypes of a foundation legume shrub drive plant–plant interactions in a Mediterranean mountain. Journal of Vegetation Science, 26, 373–384. 10.1111/jvs.12246

[ece38552-bib-0005] Alam, M. A. , Wyse, S. V. , Buckley, H. L. , Perry, G. L. , Sullivan, J. J. , Mason, N. W. , Buxton, R. , Richardson, S. J. , & Curran, T. J. (2020). Shoot flammability is decoupled from leaf flammability, but controlled by leaf functional traits. Journal of Ecology, 108(2), 641–653. 10.1111/1365-2745.13289

[ece38552-bib-0007] Anacker, B. , Rajakaruna, N. , Ackerly, D. , Harrison, S. , Keeley, J. , & Vasey, M. (2011). Ecological strategies in California chaparral: Interacting effects of soils, climate, and fire on specific leaf area. Plant Ecology and Diversity, 4(2–3), 179–188. 10.1080/17550874.2011.633573

[ece38552-bib-0008] Anderson, M. J. , Crist, T. O. , Chase, J. M. , Vellend, M. , Inouye, B. D. , Freestone, A. L. , Sanders, N. J. , Cornell, H. V. , Comita, L. S. , Davies, K. F. , Harrison, S. P. , Kraft, N. J. B. , Stegen, J. C. , & Swenson, N. G. (2011). Navigating the multiple meanings of β diversity: a roadmap for the practicing ecologist. Ecology Letters, 14, 19–28. 10.1111/j.1461-0248.2010.01552.x 21070562

[ece38552-bib-0009] Arnan, X. , Rodrigo, A. , & Retana, J. (2007). Post‐fire regeneration of Mediterranean plant communities at a regional scale is dependent on vegetation type and dryness. Journal of Vegetation Science, 18(1), 111–122. 10.1111/j.1654-1103.2007.tb02521.x

[ece38552-bib-0010] Arzani, H. , & Abedi, M. (2015). Rangeland assessment: Vegetation measurements (305 p.). University of Tehran Press.

[ece38552-bib-0011] Bahalkeh, K. , Abedi, M. , & Dianati Tilaki, G. (2017). The effect of seasons and exposures on microhabitat modifications of *Onobrychis cornuta* cushions. Environmental Erosion Research, 6, 68–80.

[ece38552-bib-0012] Bahalkeh, K. , Abedi, M. , Tilaki, D. , & Michalet, R. (2021). Fire slightly decreases on the short‐term the competitive effects of a thorny cushion shrub in a semi‐arid mountain steppe. Applied Vegetation Science, 24, e12575. 10.1111/avsc.12575

[ece38552-bib-0013] Bahalkeh, K. , Abedi, M. , & Tilaki, G. A. (2018). Effects of fire on biotic interaction of *Onobrychis cornuta* in mountain grasslands. Iranian Journal of Range and Desert Research, 25, 140–151.

[ece38552-bib-0014] Baselga, A. (2010). Partitioning the turnover and nestedness components of beta diversity. Global Ecology and Biogeography, 19, 134–143. 10.1111/j.1466-8238.2009.00490.x

[ece38552-bib-0015] Baselga, A. , Orme, D. , Villeger, S. , De Bortoli, J. , Leprieur, F. , & Baselga, M. A. (2018). Package ‘betapart’. Partitioning beta diversity into turnover and nestedness components. Version 1. https://cran.r‐project.org/package=betapart

[ece38552-bib-0016] Basu, S. , Ramegowda, V. , Kumar, A. , & Pereira, A. (2016). Plant adaptation to drought stress. F1000Research, 5, 1554. 10.12688/f1000research.7678.1 PMC4937719 27441087

[ece38552-bib-0017] Billings, W. D. (1974). Adaptations and origins of alpine plants. Arctic and Alpine Research, 6, 129–142. 10.2307/1550081

[ece38552-bib-0018] Bishop, T. R. , Robertson, M. P. , van Rensburg, B. J. , & Parr, C. L. (2015). Contrasting species and functional beta diversity in montane ant assemblages. Journal of Biogeography, 42, 1776–1786. 10.1111/jbi.12537 27563167 PMC4979679

[ece38552-bib-0019] Bond, W. , & Midgley, J. J. (1995). Kill thy neighbour: An individualistic argument for the evolution of flammability. Oikos, 73, 79–85. 10.2307/3545728

[ece38552-bib-0095] Bond, W. J. , & Midgley, J. J. (2003). The evolutionary ecology of sprouting in woody plants. International Journal of Plant Sciences, 164(S3), S103–S114.

[ece38552-bib-0020] Bowd, E. J. , Lindenmayer, D. B. , Banks, S. C. , & Blair, D. P. (2018). Logging and fire regimes alter plant communities. Ecological Applications: A Publication of the Ecological Society of America, 28, 826–841. 10.1002/eap.1693 29411919

[ece38552-bib-0021] Bowman, D. M. , Perry, G. L. , Higgins, S. I. , Johnson, C. N. , Fuhlendorf, S. D. , & Murphy, B. P. (2016). Pyrodiversity is the coupling of biodiversity and fire regimes in food webs. Philosophical Transactions of the Royal Society B: Biological Sciences, 371, 20150169. 10.1098/rstb.2015.0169 PMC4874407 27216526

[ece38552-bib-0022] Buhk, C. , Meyn, A. , & Jentsch, A. (2007). The challenge of plant regeneration after fire in the Mediterranean Basin: Scientific gaps in our knowledge on plant strategies and evolution of traits. Plant Ecology, 192(1), 1–19. 10.1007/s11258-006-9224-2

[ece38552-bib-0023] Bureau of Environment in Golestan Province . (2014). Golestan National Park fire report. Bureau of Environment in Golestan Province.

[ece38552-bib-0024] Bytebier, B. , Antonelli, A. , Bellstedt, D. U. , & Linder, H. P. (2010). Estimating the age of fire in the Cape flora of South Africa from an orchid phylogeny. Proceedings of the Royal Society B: Biological Sciences, 278, 188–195. 10.1098/rspb.2010.1035 PMC3013384 20685712

[ece38552-bib-0025] Calitz, W. , Potts, A. J. , & Cowling, R. M. (2015). Investigating species‐level flammability across five biomes in the Eastern Cape, South Africa. South African Journal of Botany, 101, 32–39.

[ece38552-bib-0026] Calvo, L. , Baeza, J. , Marcos, E. , Santana, V. , & Papanastasis, V. P. (2012). Post‐fire management of shrublands. In F. Moreira , M. Arianoutsou , P. Corona , & J. De las Heras (Eds.), Post‐fire management and restoration of Southern European Forests (pp. 293–319). Springer.

[ece38552-bib-0027] Cornelissen, J. H. C. , Lavorel, S. , Garnier, E. , Díaz, S. , Buchmann, N. , Gurvich, D. E. , Reich, P. B. , Steege, H. T. , Morgan, H. D. , Heijden, M. G. A. V. D. , Pausas, J. G. , & Poorter, H. (2003). A handbook of protocols for standardised and easy measurement of plant functional traits worldwide. Australian Journal of Botany, 51, 335–380. 10.1071/BT02124

[ece38552-bib-0028] Crist, T. O. , Veech, J. A. , Gering, J. C. , & Summerville, K. S. (2003). Partitioning species diversity across landscapes and regions: a hierarchical analysis of α, β, and γ diversity. The American Naturalist, 162, 734–743. 10.1086/378901 14737711

[ece38552-bib-0029] Davies, K. W. , Svejcar, T. J. , & Bates, J. D. (2009). Interaction of historical and nonhistorical disturbances maintains native plant communities. Ecological Applications, 19, 1536–1545. 10.1890/09-0111.1 19769101

[ece38552-bib-0030] Díaz, S. , Kattge, J. , Cornelissen, J. H. , Wright, I. J. , Lavorel, S. , Dray, S. , Reu, B. , Kleyer, M. , Wirth, C. , Prentice, I. C. , & Garnier, E. (2016). The global spectrum of plant form and function. Nature, 529(7585), 167–171. 26700811 10.1038/nature16489

[ece38552-bib-0031] Estes, B. L. , Knapp, E. E. , Skinner, C. N. , Miller, J. D. , & Preisler, H. K. (2017). Factors influencing fire severity under moderate burning conditions in the Klamath Mountains, northern California, USA. Ecosphere, 8, e01794. 10.1002/ecs2.1794

[ece38552-bib-0032] Evans, J. P. (2010). Global warming impact on the dominant precipitation processes in the Middle East. Theoretical and Applied Climatology, 99(3), 389–402. 10.1007/s00704-009-0151-8

[ece38552-bib-0033] Feizabadi, M. F. , Tahmasebi, P. , Broujeni, E. A. , Ebrahimi, A. , & Omidipour, R. (2021). Functional diversity, functional composition and functional β diversity drive aboveground biomass across different bioclimatic rangelands. Basic and Applied Ecology, 52, 68–81. 10.1016/j.baae.2021.01.007

[ece38552-bib-0096] Fernandes, P. M. , Vega, J. A. , Jimenez, E. , & Rigolot, E. (2008). Fire resistance of European pines. Forest Ecology and Management, 256(3), 246–255.

[ece38552-bib-0034] Gómez‐González, S. , Torres‐Díaz, C. , Bustos‐Schindler, C. , & Gianoli, E. (2011). Anthropogenic fire drives the evolution of seed traits. Proceedings of the National Academy of Sciences of the United States of America, 108, 18743–18747. 10.1073/pnas.1108863108 22065739 PMC3219139

[ece38552-bib-0035] Gotelli, N. J. , & Colwell, R. K. (2001). Quantifying biodiversity: Procedures and pitfalls in the measurement and comparison of species richness. Ecology Letters, 4(4), 379–391. 10.1046/j.1461-0248.2001.00230.x

[ece38552-bib-0036] Grime, J. P. (1973). Competitive exclusion in herbaceous vegetation. Nature, 242(5396), 344–347.

[ece38552-bib-0037] Grimm, N. B. , Chapin, F. S. , Bierwagen, B. , Gonzalez, P. , Groffman, P. M. , Luo, Y. , Melton, F. , Nadelhoffer, K. , Pairis, A. , Raymond, P. A. , Schimel, J. , & Williamson, C. E. (2013). The impacts of climate change on ecosystem structure and function. Frontiers in Ecology and the Environment, 11(9), 474–482. 10.1890/120282

[ece38552-bib-0038] Gross, N. , Börger, L. , Duncan, R. P. , & Hulme, P. E. (2013). Functional differences between alien and native species: do biotic interactions determine the functional structure of highly invaded grasslands? Functional Ecology, 27(5), 1262–1272. 10.1111/1365-2435.12120

[ece38552-bib-0039] Hart, S. J. , Henkelman, J. , McLoughlin, P. D. , Nielsen, S. E. , Truchon‐Savard, A. , & Johnstone, J. F. (2019). Examining forest resilience to changing fire frequency in a fire‐prone region of boreal forest. Global Change Biology, 25(3), 869–884. 10.1111/gcb.14550 30570807

[ece38552-bib-0040] He, T. , Lamont, B. B. , & Pausas, J. G. (2019). Fire as a key driver of Earth's biodiversity. Biological Reviews, 94(6), 1983–2010. 10.1111/brv.12544 31298472

[ece38552-bib-0041] He, T. , Pausas, J. G. , Belcher, C. M. , Schwilk, D. W. , & Lamont, B. B. (2012). Fire‐adapted traits of Pinus arose in the fiery Cretaceous. New Phytologist, 194, 751–759. 10.1111/j.1469-8137.2012.04079.x 22348443

[ece38552-bib-0042] Hernández‐Serrano, A. , Verdú, M. , González‐Martínez, S. C. , & Pausas, J. G. (2013). Fire structures pine serotiny at different scales. American Journal of Botany, 100, 2349–2356. 10.3732/ajb.1300182 24222682

[ece38552-bib-0043] Heydari, M. , Aazami, F. , Faramarzi, M. , Omidipour, R. , Bazgir, M. , Pothier, D. , & Prévosto, B. (2019). Interaction between climate and management on beta diversity components of vegetation in relation to soil properties in arid and semi‐arid oak forests, Iran. Journal of Arid Land, 11(1), 43–57. 10.1007/s40333-018-0024-z

[ece38552-bib-0044] Heydari, M. , Moradizadeh, H. , Omidipour, R. , Mezbani, A. , & Pothier, D. (2020). Spatio‐temporal changes in the understory heterogeneity, diversity, and composition after fires of different severities in a semiarid oak (*Quercus brantii* Lindl.) forest. Land Degradation & Development, 31(8), 1039–1049. 10.1002/ldr.3518

[ece38552-bib-0045] Heydari, M. , Omidipour, R. , Abedi, M. , & Baskin, C. (2017). Effects of fire disturbance on alpha and beta diversity and on beta diversity components of soil seed banks and aboveground vegetation. Plant Ecology and Evolution, 150, 247–256. 10.5091/plecevo.2017.1344

[ece38552-bib-0046] Holden, Z. A. , Morgan, P. , & Evans, J. S. (2009). A predictive model of burn severity based on 20‐year satellite‐inferred burn severity data in a large southwestern US wilderness area. Forest Ecology and Management, 258, 2399–2406. 10.1016/j.foreco.2009.08.017

[ece38552-bib-0047] Hurteau, M. D. , Bradford, J. B. , Fulé, P. Z. , Taylor, A. H. , & Martin, K. L. (2014). Climate change, fire management, and ecological services in the southwestern US. Forest Ecology and Management, 327, 280–289. 10.1016/j.foreco.2013.08.007

[ece38552-bib-0048] Jahdi, R. , Salis, M. , Darvishsefat, A. A. , Alcasena, F. , Mostafavi, M. A. , Etemad, V. , Lozano, O. M. , & Spano, D. (2016). Evaluating fire modelling systems in recent wildfires of the Golestan National Park, Iran. Forestry: An International Journal of Forest Research, 89, 136–149. 10.1093/forestry/cpv045

[ece38552-bib-0049] Kasischke, E. S. , Christensen, N. L. Jr , & Stocks, B. J. (1995). Fire, global warming, and the carbon balance of boreal forests. Ecological Applications, 5(2), 437–451. 10.2307/1942034

[ece38552-bib-0050] Keeley, J. E. (2009). Fire intensity, fire severity and burn severity: A brief review and suggested usage. International Journal of Wildland Fire, 18(1), 116–126. 10.1071/WF07049

[ece38552-bib-0051] Keeley, J. E. , Lubin, D. , & Fotheringham, C. J. (2003). Fire and grazing impacts on plant diversity and alien plant invasions in the southern Sierra Nevada. Ecological Applications, 13(5), 1355–1374. 10.1890/02-5002

[ece38552-bib-0052] Keeley, J. E. , Pausas, J. G. , Rundel, P. W. , Bond, W. J. , & Bradstock, R. A. (2011). Fire as an evolutionary pressure shaping plant traits. Trends in Plant Science, 16, 406–411. 10.1016/j.tplants.2011.04.002 21571573

[ece38552-bib-0053] Khaled, R. A. H. , Duru, M. , Decruyenaere, V. , Jouany, C. , & Cruz, P. (2006). Using leaf traits to rank native grasses according to their nutritive value. Rangeland Ecology and Management, 59, 648–654. 10.2111/05-031R2.1

[ece38552-bib-0054] Krawchuk, M. A. , Moritz, M. A. , Parisien, M. A. , Van Dorn, J. , & Hayhoe, K. (2009). Global pyrogeography: The current and future distribution of wildfire. PLoS One, 4, e5102. 10.1371/journal.pone.0005102 19352494 PMC2662419

[ece38552-bib-0055] Laliberté, E. , & Legendre, P. (2010). A distance‐based framework for measuring functional diversity from multiple traits. Ecology, 91, 299–305. 10.1890/08-2244.1 20380219

[ece38552-bib-0056] Laliberté, E. , Legendre, P. , & Shipley, B. (2015). Package ‘FD’: Measuring functional diversity from multiple traits, and other tools for functional ecology, Version 1.0‐12. https://cran.r‐project.org/package=FD 10.1890/08-2244.1 20380219

[ece38552-bib-0057] Lambers, H. , Chapin, F. S. III , & Pons, T. L. (2008). Plant physiological ecology. Springer Science & Business Media.

[ece38552-bib-0058] Lande, R. (1996). Statistics and partitioning of species diversity, and similarity among multiple communities. Oikos, 76(1), 5–13. 10.2307/3545743

[ece38552-bib-0059] Lavorel, S. , Díaz, S. , Cornelissen, J. H. C. , Garnier, E. , Harrison, S. P. , McIntyre, S. , Pausas, J. G. , Pérez‐Harguindeguy, N. , Roumet, C. , & Urcelay, C. (2007). Plant functional types: are we getting any closer to the Holy Grail? In J. Canadell , L. F. Pitelka , & D. Pataki (Eds.), Terrestrial ecosystems in a changing world (pp. 149–164). Springer.

[ece38552-bib-0060] Li, Y. U. , Dong, S. , Gao, Q. , Zhang, Y. , Liu, S. , Swift, D. , Zhao, J. , Ganjurjav, H. , Hu, G. , Wang, X. , Yan, Y. , Cao, X. , Li, W. , Luo, W. , Zhao, Z. , Li, S. , & Gao, X. (2019). Grazing promotes plant functional diversity in alpine meadows on the Qinghai‐Tibetan Plateau. The Rangeland Journal, 41, 73–81. 10.1071/RJ18091

[ece38552-bib-0061] Lima, C. H. , AghaKouchak, A. , & Randerson, J. T. (2018). Unraveling the role of temperature and rainfall on active fires in the Brazilian Amazon using a nonlinear Poisson model. Journal of Geophysical Research: Biogeosciences, 123, 117–128. 10.1002/2017JG003836

[ece38552-bib-0062] Madadgar, S. , Sadegh, M. , Chiang, F. , Ragno, E. , & AghaKouchak, A. (2020). Quantifying increased fire risk in California in response to different levels of warming and drying. Stochastic Environmental Research and Risk Assessment, 34, 2023–2031.

[ece38552-bib-0063] Mason, N. W. , Mouillot, D. , Lee, W. G. , & Wilson, J. B. (2005). Functional richness, functional evenness and functional divergence: The primary components of functional diversity. Oikos, 111, 112–118. 10.1111/j.0030-1299.2005.13886.x

[ece38552-bib-0064] McLauchlan, K. K. , Higuera, P. E. , Miesel, J. , Rogers, B. M. , Schweitzer, J. , Shuman, J. K. , Tepley, A. J. , Varner, J. M. , Veblen, T. T. , Adalsteinsson, S. A. , Balch, J. K. , Baker, P. , Batllori, E. , Bigio, E. , Brando, P. , Cattau, M. , Chipman, M. L. , Coen, J. , Crandall, R. , … Watts, A. C. (2020). Fire as a fundamental ecological process: Research advances and frontiers. Journal of Ecology, 108(5), 2047–2069. 10.1111/1365-2745.13403

[ece38552-bib-0065] Memariani, F. , Zarrinpour, V. , & Akhani, H. (2016). A review of plant diversity, vegetation, and phytogeography of the Khorassan‐Kopet Dagh floristic province in the Irano‐Turanian region (northeastern Iran–southern Turkmenistan). Phytotaxa, 249, 8–30. 10.11646/phytotaxa.249.1.4

[ece38552-bib-0066] Moradizadeh, H. , Heydari, M. , Omidipour, R. , Mezbani, A. , & Prévosto, B. (2020). Ecological effects of fire severity and time since fire on the diversity partitioning, composition and niche apportionment models of post‐fire understory vegetation in semi‐arid oak forests of Western Iran. Ecological Engineering, 143, 105694. 10.1016/j.ecoleng.2019.105694

[ece38552-bib-0067] Mouillot, D. , Graham, N. A. , Villéger, S. , Mason, N. W. , & Bellwood, D. R. (2013). A functional approach reveals community responses to disturbances. Trends in Ecology & Evolution, 28(3), 167–177. 23141923 10.1016/j.tree.2012.10.004

[ece38552-bib-0068] Omidipour, R. , Tahmasebi, P. , Faizabadi, M. F. , Faramarzi, M. , & Ebrahimi, A. (2021). Does β diversity predict ecosystem productivity better than species diversity? Ecological Indicators, 122, 107212. 10.1016/j.ecolind.2020.107212

[ece38552-bib-0069] Pastro, L. A. , Dickman, C. R. , & Letnic, M. (2014). Fire type and hemisphere determine the effects of fire on the alpha and beta diversity of vertebrates: A global meta‐analysis. Global Ecology and Biogeography, 23, 1146–1156. 10.1111/geb.12195

[ece38552-bib-0070] Pausas, J. G. , Bradstock, R. A. , Keith, D. A. , & Keeley, J. E. (2004). Plant functional traits in relation to fire in crown‐fire ecosystems. Ecology, 85, 1085–1100. 10.1890/02-4094

[ece38552-bib-0071] Pausas, J. G. , & Ribeiro, E. (2013). The global fire–productivity relationship. Global Ecology and Biogeography, 22, 728–736. 10.1111/geb.12043

[ece38552-bib-0072] Pausas, J. G. , & Verdú, M. (2008). Fire reduces morphospace occupation in plant communities. Ecology, 89(8), 2181–2186. 18724728 10.1890/07-1737.1

[ece38552-bib-0073] Pierce, S. M. , Esler, K. , & Cowling, R. M. (1995). Smoke‐induced germination of succulents (Mesembryanthemaceae) from fire‐prone and fire‐free habitats in South Africa. Oecologia, 102, 520–522. 10.1007/BF00341366 28306897

[ece38552-bib-0074] Polley, H. W. , Briske, D. D. , Morgan, J. A. , Wolter, K. , Bailey, D. W. , & Brown, J. R. (2013). Climate change and North American rangelands: Trends, projections, and implications. Rangeland Ecology & Management, 66(5), 493–511. 10.2111/REM-D-12-00068.1

[ece38552-bib-0075] R Core Team . (2018). R: A language and environment for statistical computing. R Foundation for Statistical Computing. Retrieved from https://www.R‐project.org/

[ece38552-bib-0076] Rao, C. R. (1982). Diversity and dissimilarity coefficients: A unified approach. Theoretical Population Biology, 21, 24–43. 10.1016/0040-5809(82)90004-1

[ece38552-bib-0077] Rhodes, E. C. , Bates, J. D. , Sharp, R. N. , & Davies, K. W. (2010). Fire effects on cover and dietary resources of sage‐grouse habitat. The Journal of Wildlife Management, 74, 755–764. 10.2193/2009-143

[ece38552-bib-0078] Ricotta, C. , & Burrascano, S. (2008). Beta diversity for functional ecology. Preslia, 80(1), 61–72.

[ece38552-bib-0079] Roshan, G. , Ghanghermeh, A. , Nasrabadi, T. , & Meimandi, J. B. (2013). Effect of global warming on intensity and frequency curves of precipitation, case study of Northwestern Iran. Water Resources Management, 27(5), 1563–1579. 10.1007/s11269-013-0258-7

[ece38552-bib-0080] Safaian, N. , Shokri, M. , Ahmadi, M. Z. , Atrakchali, A. , & Tavili, A. (2005). Fire influence on the grassland vegetation in Golestan National Park (Alborz MTS. Iran). Polish Journal of Ecology, 53, 435–443.

[ece38552-bib-0081] Sakschewski, B. , von Bloh, W. , Boit, A. , Rammig, A. , Kattge, J. , Poorter, L. , Peñuelas, J. , & Thonicke, K. (2015). Leaf and stem economics spectra drive diversity of functional plant traits in a dynamic global vegetation model. Global Change Biology, 21(7), 2711–2725. 10.1111/gcb.12870 25611734

[ece38552-bib-0082] Simpson, K. J. , Ripley, B. S. , Christin, P. A. , Belcher, C. M. , Lehmann, C. E. , Thomas, G. H. , & Osborne, C. P. (2016). Determinants of flammability in savanna grass species. Journal of Ecology, 104(1), 138–148. 10.1111/1365-2745.12503 PMC4738432 26877549

[ece38552-bib-0084] Swenson, N. G. , Anglada‐Cordero, P. , & Barone, J. A. (2011). Deterministic tropical tree community turnover: Evidence from patterns of functional beta diversity along an elevational gradient. Proceedings of the Royal Society B: Biological Sciences, 278(1707), 877–884. 10.1098/rspb.2010.1369 PMC3049044 20861048

[ece38552-bib-0085] Tahmasebi, P. , Mehdi, M. , & Omidipour, R. (2017). Plant functional identity as the predictor of carbon storage in semi‐arid ecosystems. Plant Ecology and Diversity, 10, 139–151. 10.1080/17550874.2017.1355414

[ece38552-bib-0087] Tuomisto, H. (2010). A diversity of beta diversities: Straightening up a concept gone awry. Part 1. Defining beta diversity as a function of alpha and gamma diversity. Ecography, 33, 2–22. 10.1111/j.1600-0587.2009.05880.x

[ece38552-bib-0088] Villéger, S. , Grenouillet, G. , & Brosse, S. (2013). Decomposing functional β‐diversity reveals that low functional β‐diversity is driven by low functional turnover in European fish assemblages. Global Ecology and Biogeography, 22, 671–681. 10.1111/geb.12021

[ece38552-bib-0089] Villéger, S. , Mason, N. W. , & Mouillot, D. (2008). New multidimensional functional diversity indices for a multifaceted framework in functional ecology. Ecology, 89, 2290–2301. 10.1890/07-1206.1 18724739

[ece38552-bib-0090] Violle, C. , Thuiller, W. , Mouquet, N. , Munoz, F. , Kraft, N. J. , Cadotte, M. W. , & Mouillot, D. (2017). Functional rarity: The ecology of outliers. Trends in Ecology & Evolution, 32(5), 356–367. 28389103 10.1016/j.tree.2017.02.002 PMC5489079

[ece38552-bib-0094] Violle, C. , Navas, M. L. , Vile, D. , Kazakou, E. , Fortunel, C. , Hummel, I. , & Garnier, E. (2007). Let the concept of trait be functional! Oikos, 116(5), 882–892.

[ece38552-bib-0091] Westoby, M. , Falster, D. S. , Moles, A. T. , Vesk, P. A. , & Wright, I. J. (2002). Plant ecological strategies: Some leading dimensions of variation between species. Annual Review of Ecology and Systematics, 33, 125–159. 10.1146/annurev.ecolsys.33.010802.150452

[ece38552-bib-0097] Whittaker, R. H. (1960). Vegetation of the Siskiyou mountains, Oregon and California. Ecological Monographs, 30(3), 279–338.

[ece38552-bib-0092] Wright, I. J. , Reich, P. B. , Westoby, M. , Ackerly, D. D. , Baruch, Z. , Bongers, F. , Cavender‐Bares, J. , Chapin, T. , Cornelissen, J. H. C. , Diemer, M. , Flexas, J. , Garnier, E. , Groom, P. K. , Gulias, J. , Hikosaka, K. , Lamont, B. B. , Lee, T. , Lee, W. , Lusk, C. , … Villar, R. (2004). The worldwide leaf economics spectrum. Nature, 428, 821. 10.1038/nature02403 15103368

[ece38552-bib-0093] Xue, R. , Yang, Q. , Miao, F. , Wang, X. , & Shen, Y. (2018). Slope aspect influences plant biomass, soil properties and microbial composition in alpine meadow on the Qinghai‐Tibetan plateau. Journal of Soil Science and Plant Nutrition, 18, 1–12. 10.4067/S0718-95162018005000101

