# Corrigendum

**DOI:** 10.1002/ece3.9841

**Published:** 2023-02-26

In the article by Abedi et al. (2022), the name of the third author was incorrect. The article has been corrected online, so author byline now reads as:

Mehdi Abedi | Reza Omidipour | Seyed **Vrya** Hosseini | Khadijeh Bahalkeh | Nicolas Gross

The authors apologize for the error.
