# Supplementary material for: Fire disturbance effects on plant taxonomic and functional β‐diversity mediated by topographic exposure
Source: Ecol Evol. 2022 Jan 25;12(1):e8552. doi: 10.1002/ece3.8552 (PMC8796949; doi:10.1002/ece3.8552)
Supplement: Supplementary file 1 — Supplementary Material [file ECE3-12-e8552-s001.docx]

**Table S1-** Cover percentage (n=20) for different functional groups in four study sites.

|  | **North-Fire** | **South-Fire** | **North-Control** | **South-Control** |
| --- | --- | --- | --- | --- |
| **Annuals** | 0.075 | 0.11 | 0.09 | 0.075 |
| **Perennial grass** | 11.54 | 9.37 | 30.70 | 11.54 |
| **Perennial herb** | 12.66 | 27.24 | 26.20 | 12.66 |
| **Shrubs** | 7.19 | 3.48 | 24.61 | 7.19 |
| **Sub shrubs** | 2.63 | 1.135 | 5.33 | 2.625 |
| **Geophytes** | 0.015 | 0.005 | 0.025 | 0.015 |
| **Total cover** | 34.11 | 41.33 | 86.95 | 34.11 |

| 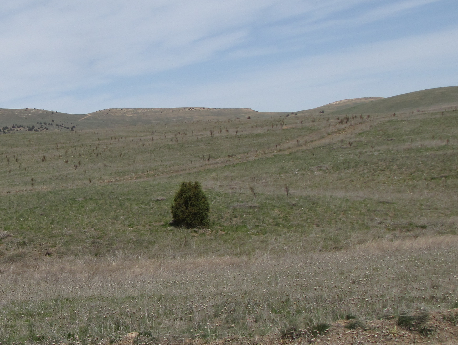 | 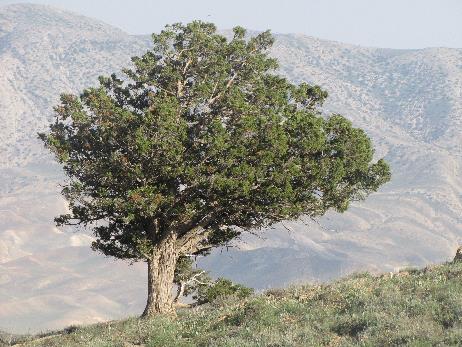 |
| --- | --- |
| Young Juniper shrubs close to north control sites | Old Juniper shrubs in montain grasslands |
| 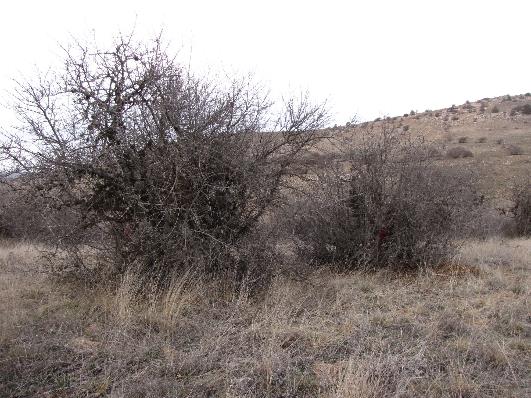 | 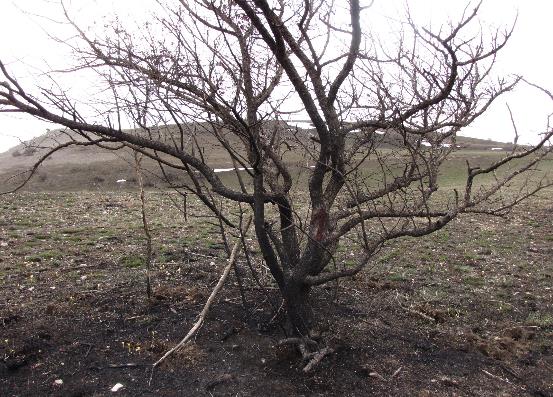 |
| Acer shrubs in south control site at 2013 | Burned Acer shrubs in the south burned site at 2013 |
| 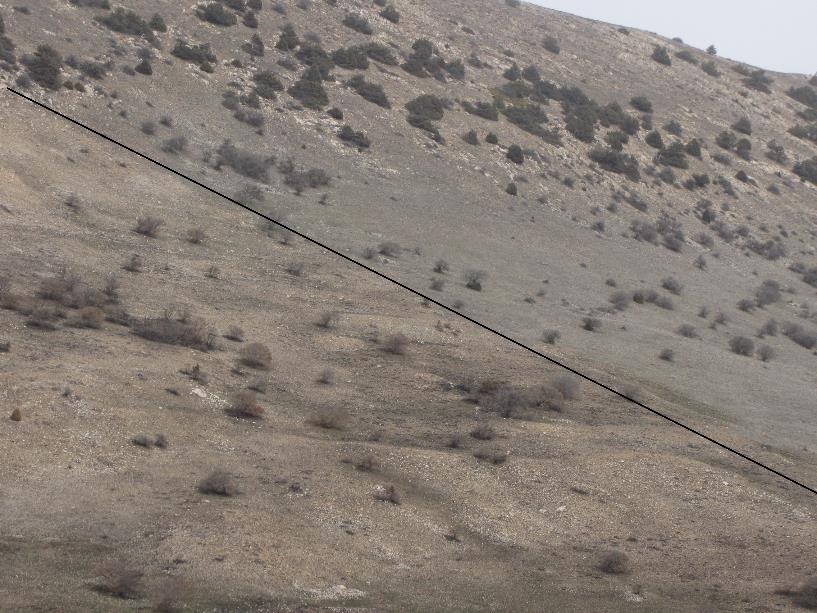 | |
| **Figure S1.** Burned (left) and control (right) sites in the south exposure. In both sites, woody shrubs including Acer and Junipers could be observed at 2013. This confirm that there were no fire for several decades. Both Acer and Juniper are fire sensitive and will be killed after burning. Observing Matured shrubs indicated there were no fire for several decades. This results also confirmed by fire report of golestan national park. | |
